# Sick Children Crying for Help: Fostering Adverse Event Reports

**DOI:** 10.1371/journal.pmed.1002216

**Published:** 2017-01-17

**Authors:** Gordon D. Schiff

**Affiliations:** 1 Center for Patient Safety Research and Practice, Division of General Internal Medicine, Brigham and Women's Hospital, Boston, Massachusetts, United States of America; 2 Harvard Medical School Center for Primary Care, Boston, Massachusetts, United States of America

## Abstract

In a Perspective, Gordon Schiff discusses the importance of appropriately analyzing adverse event reports.

In this week’s *PLOS Medicine*, Philippa Rees and a team of patient safety experts delved into a decade of reports to the United Kingdom National Reporting and Learning System to cull more than 2,000 events occurring in sick children [1]. Their retrospective finding of key areas of outpatient harm and risk points to a number of specific areas for improvement. The study is valuable both for the specific findings, lessons, and insights and also for encouraging us to grapple with the value of such reporting systems, analysis of collected reports, and ways of better leveraging findings to prevent harm in the future. Representative of the state of the art in incident reporting and review, there is cause for celebration as well as concern—celebration because assembling a large number of case reports allows us to step back and get beyond these single/isolated/disconnected reports to see a bigger picture, and concern because one has to worry that two decades of such efforts to collect, classify, draw conclusions, and effectively stimulate corrective changes from adverse events has resulted in a paucity of measurable benefits, with considerable wasted effort and lost opportunities for meaningful learning and improvement [2–4].

While not a pediatrician, as a primary care physician and patient safety researcher I have spent considerable time both submitting and reviewing safety reports [5,6]. At one point, I had filed more error and adverse drug reactions reports than all the other physicians at my public hospital in Chicago combined, making me either the institution’s most dangerous prescriber or its most diligent reporter [7]. Hoping it is more the latter, it is sobering to consider how infrequently adverse events and errors are being reported. Not only are we missing many adverse events, but those being reported likely are not perfectly representative of all errors that are occurring [8]. Thus, it is neither advisable nor fair to use report rates (or changes in rates) as measures of the epidemiology of quality or improvement efforts. Thus, I would caution readers to be wary of accepting the authors’ opening suggestion that we can correlate these reported safety issues with poorer outcome measures or higher mortality rates of the United Kingdom relative to other European countries.

But what about the reports themselves—this wealth of rich case examples of actual problems transmitted straight from the front lines? We certainly have a debt to those who took the time and effort and perhaps even took risks to report these adverse events and owe them (as well as patients who may have been harmed) meaningful follow-up, learning, feedback, sharing, and improvement. In-depth examination both horizontally (to connect the reports with each other to see aggregate data and trends) and vertically (to dig deeper, delving into the rich details of the free-text narrative details that accompany a good report) as the authors have done is needed, yet is more often the exception than the rule. While I am not aware of any studies that have quantified the extent of this failure, this waste, and these wasted opportunities, I suspect it is huge.

A key aspect of meaningfully bringing together these reports is classification of the events. Thinking critically about this step is necessary to avoid compounding empty collecting exercises with pointless taxonomy counting rituals. Analysis should breathe additional life into safety reports, rather than simply “putting them to bed.” What does a vision of such breathing life look and feel like, and to what extent does the Rees et al. work give us a glimpse of it?

Two key ingredients in my view are (1) careful, timely review and contemplation of report narratives and (2) envisioning the ways such an event could happen elsewhere with an eye to error-proofing redesign. Basic quality improvement conceptual tools such as a) the “5 Whys” (digging progressively deeper by asking “why” five times to get to the root of underlying contributing causes) [9], b) distinguishing “special cause” from “common cause” (a statistical process control tool begging for more use in health care), c) minimizing “tampering” (well-meaning attempts to make changes that can introduce more variation and quality problems), and d) avoiding “suboptimization” (another improvement pitfall whereby changes are recommended or made that may help a narrowly conceived problem but create a more complex and dysfunctional system overall) need to be applied more regularly and rigorously [10,11].

With this perspective, how does the study by Rees and colleagues help move us forward? One way is by shining a light on two somewhat new or mostly untapped venues for collecting errors and adverse event reports—community pharmacies and telephone triage call centers. These two settings led the pack in reports of issues, which originated from and illustrated a number of vulnerabilities of special relevance to a pediatric population—particularly, special dosing/dispensing considerations (often requiring individualized medication compounding) and delays in recognizing septicemia. Also noteworthy was the finding that diagnostic delays had the highest burden of harm, something we have also argued and seen in malpractice cases [12]. Diagnostic errors are relatively infrequently reported to adverse event reporting systems, so finding substantial numbers here suggests this is the tip of a larger iceberg [13].

In their list of contributing causes, one item jarringly stands out both for its frequency and disharmony with a systems and just culture perspective: “failure to follow protocol” [14]. Were such reports perhaps more akin to incident reports submitted by supervisors to “write up” an employee who may have committed an error as documentation for the employee’s personnel record and as a warning? To the extent these reports were grounded in a retributive workplace culture, rather than a more ideal model of frontline staff submitting reports of errors or problems that they had seen, been involved with, or personally committed, based on caring deeply about the need to share these widely to help others avoid such pitfalls, these reports fulfill a less noble and valuable function.

Lest we throw out the babies with the bathwater, we need to listen carefully to these incidents, analyze them, and act on them better (Table 1). Safety experts are debating and rethinking incident reporting on multiple continents [4,15]. Meanwhile, there is much to be learned from these reports, and the paper by Rees and colleagues just scratches the surface. Quality and learning and improvement from incident reporting need to be supported and enhanced at every step, but currently, there is a paucity of resources, responsiveness, and responsibility to do this well [16,17]. For these children and their parents, each institution should use these reports to ask and examine the questions: is this happening here; if so, how often; and how can we work at the front line and at the larger health authority level to make sure such incidents are less likely in the future [16]?

**Table 1:** Framework for Nurturing Reporting: Opportunities/Imperatives for Incident Reporting Improvement.

**Front End: Detecting and Reporting**
*Buy-in*: to motivate engagement, making effort
*Noticing*: being alert to everyday errors, large and small; process; and outcome
*Streamlined* automated reporting structures and processes to make it easy to file reports and to capture relevant data/narrative
*Avoiding blame* and fear
*Creating a culture* where everyone views sharing problems as fun, fundamental, and integral to work
**Review: Investigation and Analysis**
*Classifying*: availability/use of parsimonious, rich, and reliable taxonomies
Delving deeper into *narratives*; coding themes
Meaningful additional *investigation* of the case/issues, including (where appropriate) reaching out to frontline staff for additional input
Connecting across cases to *identify patterns*
Hypothesizing and proposing ways to *prevent*
Statistical; *data mining* sweeps through reports and narratives
**Back End: Learning and Improvement**
Differentiating system/systematic (*common cause)* versus outlier (*special cause)* types of adverse events
Formulating, consensus process for *recommendations*, improvements to test
Widespread c*ommunication* of findings
*Integrating awareness* of the risk (situational awareness) into daily practice, workflow (visual cues and CDS)
*Feeding back* findings directly to reporting staff/institutions and *hearing back* from them regarding further follow-up and response to recommendations
*Revisiting* issues; monitoring over time

Abbreviations: CDS, clinical decision support
